# Exterpation of Diseased Eye to Preserve Healthy One

**Published:** 1862-09

**Authors:** 


Exterpation of Diseased Eye to Preserve Healthy one.—The practice of extirpating a diseased eye to preserve a healthy one is disapproved of by Sichel, the French oculist, The following are stated to be his views:

"With regard to the extirpation of a diseased eye, practiced for the purpose of preserving the sound one, M. Sichel says that it is an operation which has been greatly abused of late. It is undoubtedly true that a disease in one eye may at least effect the healthy eye; but affections of this nature very often depend upon constitutional causes; and, when this is the case, it happens that the healthy eye becomes at last affected as the result of the diseased constitutional conditions. The extirpation of the eye primitively affected cannot in any way cut short the influence of these constitutional causes upon the healthy eye. The general affection of the body was the original cause of the occular disease, and is also the cause which keeps up the disease; and the right treatment therefore, for the preservation of the healthy one, is to attack by therapeutics the general affections. M. Sichel also remarked, that his observations of the good effects of general treatment in diseases of the eye had often led him to combat the too frequent tendency to surgical operations in these diseases."

---

A case was recently tried in England, involving the question of the proper disposition of the "dissected fragments of humanity, as they pass from the schools to the burial-ground." The law requires that the bodies of dissected persons shall be "decently interred in consecrated ground."—

Counsel contended that "inasmuch as the burial service was intended as a solemn rite for the consolation and benefit of the living, its performance in the absence of all witnesses over the decayed fragments of a dissected body was improper and unbecoming." The judge, however, overruled this opinion, and decided that the remains "should have the burial service read over them."—*Ibid.*

